# Profiling of Blood-Brain Barrier Disruption in Mouse Intracerebral Hemorrhage Models: Collagenase Injection vs. Autologous Arterial Whole Blood Infusion

**DOI:** 10.3389/fncel.2021.699736

**Published:** 2021-08-26

**Authors:** Peijun Jia, Jinxin He, Zefu Li, Junmin Wang, Lin Jia, Ruochen Hao, Jonathan Lai, Weidong Zang, Xuemei Chen, Jian Wang

**Affiliations:** ^1^Department of Anatomy, College of Basic Medical Sciences, Zhengzhou University, Zhengzhou, China; ^2^School of Life Sciences, Zhengzhou University, Zhengzhou, China; ^3^Pre-med Track Majoring in Biology, Baylor University, Waco, TX, United States

**Keywords:** intracerebral hemorrhage, collagenase, autologous blood, blood-brain barrier, tight junction, aquaporin 4, matrix metalloproteinase-9, transmission electron microscope

## Abstract

Disruption of the blood-brain barrier (BBB) and the subsequent formation of brain edema is the most severe consequence of intracerebral hemorrhage (ICH), leading to drastic neuroinflammatory responses and neuronal cell death. A better understanding of ICH pathophysiology to develop effective therapy relies on selecting appropriate animal models. The collagenase injection ICH model and the autologous arterial whole blood infusion ICH model have been developed to investigate the pathophysiology of ICH. However, it remains unclear whether the temporal progression and the underlying mechanism of BBB breakdown are similar between these two ICH models. In this study, we aimed to determine the progression and the mechanism of BBB disruption via the two commonly used murine ICH models: the collagenase-induced ICH model (c-ICH) and the double autologous whole blood ICH model (b-ICH). Intrastriatal injection of 0.05 U collagenase or 20 μL autologous blood was used for a comparable hematoma volume in these two ICH models. Then we analyzed BBB permeability using Evan’s blue and IgG extravasation, evaluated tight junction (TJ) damage by transmission electron microscope (TEM) and Western blotting, and assessed matrix metalloproteinase-9 (MMP-9) activity and aquaporin 4 (AQP4) mRNA expression by Gelatin gel zymography and RT-PCR, respectively. The results showed that the BBB leakage was associated with a decrease in TJ protein expression and an increase in MMP-9 activity and AQP4 expression on day 3 in the c-ICH model compared with that on day 5 in the b-ICH model. Additionally, using TEM, we found that the TJ was markedly damaged on day 3 in the c-ICH model compared with that on day 5 in the b-ICH model. In conclusion, the BBB was disrupted in the two ICH models; compared to the b-ICH model, the c-ICH model presented with a more pronounced disruption of BBB at earlier time points, suggesting that the c-ICH model might be a more suitable model for studying early BBB damage and protection after ICH.

## Introduction

Intracerebral hemorrhage (ICH) is the second most common subtype of stroke, and it has a high mortality and disability rate (Wang, 2010; Lan et al., 2017). The pathophysiologic mechanism of ICH is complex, with excessive neuroinflammatory responses due to the activation of immune cells as well as the release of inflammatory cytokines (Hua et al., 2020; Ren et al., 2020; Boltze et al., 2021), which lead to the degradation of the blood-brain barrier (BBB) and neuron death (Yang et al., 2017). Subsequently, BBB disruption further aggravates vasogenic brain edema, facilitates the migration of leukocytes from blood to the brain (Jiang et al., 2020), and causes an influx of potential neurotoxic components from the blood (Madangarli et al., 2019). There has been great interest in maintaining BBB integrity and inhibiting brain inflammation after ICH.

As a dynamic structure, the BBB separates peripheral circulation from the brain. The BBB is composed of brain microvascular endothelial cells (BMECs) and their tight junctions (TJs), basement membranes, pericytes, and astrocyte terminals. Severe inflammation has been shown to have a deleterious effect on the BBB (Varatharaj and Galea, 2017). Additionally, there is evidence that secondary injury induces BMEC cell death and impairment of peri-cellular TJs which result in BBB dysfunction after stroke (Wu et al., 2019).

Many preclinical and clinical research evidence has further shown the progress of pathophysiology in ICH, but truly effective clinical treatments are limited (Ren et al., 2020). The substantial gap in applying preclinical research within clinical applications has not been solved. To study the potential therapeutic targets of BBB after ICH, it is vital to establish an appropriate animal model which directly relates to clinical therapy. Collagenase ICH model (c-ICH) and autologous blood ICH model (b-ICH) are reported to be widely used animal experiment models (Wang, 2010; Liesz et al., 2011; Li and Wang, 2017). However, there are currently no studies to comprehensively compare the cellular molecular markers of BBB disruption in these two animal models of ICH. Therefore, the dynamic changes of BBB components were compared including TJs, matrix metalloproteinases 9 (MMP), and aquaporin 4 (AQP4) after ICH between the two animal models. It is our goal to discover the model most representative of the pathophysiology of ICH patients in the clinic.

The insights gained from this study will enhance our understanding of the underlying mechanism and pathologic progress of ICH, and help determine the best animal model for preclinical ICH research targeting BBB damage and protection.

## Materials and Methods

### Animals

All animal experiments followed the ARRIVE guidelines^1^. Male mice were used in this study due to the reported hormone levels of male mice, which are more stable than those of the female mice, as shown in a previous study (Hughes, 2007). Animals for each group were randomized with the website^2^. Mice that were 6 months of age are defined as adult mice according to our previous report (Wu et al., 2011). A total number of 213 C57BL/6 male mice (6-month-old, 30–35 g) were used in our study. Mice were purchased from the Beijing Vital River Laboratory Animal Technology Co., Ltd. The mice were housed in a specific pathogen-free environment with 24-h cycles of controlled temperature and relative humidity (45–55%) with normal access to standard food and water. Animals that passed away or were euthanized were excluded from the sample size. All animal experiments were approved by the Animal Ethics Committee of Zhengzhou University and performed according to national guidelines. All assessments were performed by investigators who were blinded to experimental group assignments.

### ICH Mouse Model

The procedure for the two ICH models has been described in previous publications (Wang et al., 2008; Li et al., 2017a; Zhu et al., 2018). In brief, the mice were anesthetized with isoflurane (70% N_2_O and 30% O_2_; 4% induction, 2% maintenance). The mice were then fixed in a stereotaxic instrument with a 1 cm incision made along the sagittal suture. ICH was induced by injecting collagenase VII-S (0.05 U in 0.5 μL sterile saline, Sigma, St. Louis, MO) or 20 μL of blood was drawn from the mice’s tail artery and was injected into the left striatum which coordinates were 0.5 mm anterior, 2.1 mm lateral, and 3.1 mm ventral to the bregma. For the sham operation, 0.5 μL of sterile saline was injected. The collagenase was infused with a micro-infusion pump (1 μL, HAMILTON, 80100, United States) at a constant rate of 0.1 μL/min and veinous blood was infused by a 50 μL micro-infusion pump in two-time blocks (7 μL followed by a 10-min pause and then 13 μL followed by a 10-min pause) a rate of 1 μL/min. During the operation, internal body temperature was maintained at 37.0 ± 0.5°C by the body temperature maintenance instrument (Thermo Star, 69020). Mice that died within 24 h after surgery or with a neurological deficit score <4 were excluded from the experiment. The sham mice were treated the same way but were infused with saline. The mice were allowed to recover in separate cages with access to sufficient food and water.

### Neurologic Function

The functional outcome was evaluated using neurologic deficit score (NDS), forelimb and hind limb placing test, and the corner turn test at 6 h, 12 h, days 1, 3, and 5 after ICH. In the NDS system, mice were tested by body symmetry, gait, climbing, circling behavior, front limb symmetry, and compulsory circling. Each test was graded on a scale from 0 to 4, establishing a maximum deficit score of 24 (Li et al., 2017a). Forelimb placement was evaluated by the vibrissae-elicited forelimb placing test. The mice were placed at the edge of the table, and the vibrissae on one side were brushed. The placement was quantified as a percentage of successful responses (placed the contralateral forelimb quickly on the tabletop) in 10 trials (Chang et al., 2014). Hindlimb placing was assessed with the muscle strength of the hind limbs. The animals were placed on the edge of a table and the contralateral hind limb was pulled down. Straighten the hind limbs and given scores according to the retraction time. The test was scored as follows: immediate pullback of limb = 0; delayed pullback = 1; inability to pull back = 2. The placing was quantified in 10 successful trials, and scores were quantified as the total scores of 10 trials (Zhou et al., 2013; Li et al., 2017a). For the corner turn test, the mouse was allowed to proceed into a 30° corner and was allowed to freely turn either left or right when exiting the corner. The choice of direction during 10 repeats was recorded, and the percentage of left turns was calculated (Chang et al., 2014).

### Hemorrhagic Injury Analysis

Mice were euthanized with deep anesthesia using 3% isoflurane and perfused through the left ventricle with saline followed by 4% paraformaldehyde. Coronal brain sections were stained with cresyl violet (CV for neurons, Sigma-Aldrich) or with Luxol fast blue (LFB for myelin) at 20 μM that were spaced 180 μM apart. Sections were digitized and analyzed by Image J software. The injury volume in cubic millimeters was computed by summation of the damaged areas multiplied by the interslice distance (180 μM) (Han et al., 2016; Zhu et al., 2018).

### Brain Water Content Measurement

On day 3 or 5 after ICH, mice were euthanized under deep anesthesia with 3% isoflurane and the brain was harvested and dissected into the ipsilateral, the ipsilateral, and cerebellum, which served as an internal control. The wet weight of the brain was measured immediately and the brain tissue was heated to 100°C in a drying oven for 3 or 5 days before measuring the dry weight. We determined brain edema by calculating brain water content as follows: [(wet weight - dry weight)/wet weight] × 100% (Li et al., 2017c).

### Western Blotting

On 6 h, 12 h, days 1, 3, and 5 after ICH, occluding, cadherin-10 protein expression levels were evaluated by Western blotting (WB) according to our previous studies (Zhao et al., 2015; Wang et al., 2019). The brain tissues were sampled 2–3 mm away from the hematoma and homogenized on ice ice-cold lysis buffer (RIPA: PMSF = 100:1). The total protein count was quantified by bicinchoninic acid (BCA) protein assay (PC0020, Solarbio Science & Technology Co., Ltd., Beijing). Fifty micrograms of protein sample was separated by 10% sodium dodecyl sulfate-polyacrylamide gel electrophoresis (SDS-PAGE) and transferred to polyvinylidene fluoride (PVDF) membrane. After blocking the membrane with the skimmed milk powder, it was incubated with the primary antibodies: rabbit anti-mouse occludin (1:1,000, Abcam, Cambridge, MA), rabbit anti-mouse cadherin-10 (1:1,000, Abcam, Cambridge, MA) (1:1,000, Affinity Biosciences, OH, United States), rabbit anti-mouse GAPDH (1:1,000, Abcam, Cambridge, MA) at 4°C overnight. The membranes were then incubated with HRP-labeled anti-rabbit secondary antibodies (1:10,000, Santa Cruz, Dallas, TX) at room temperature for 2 h. The blots were detected with the Fluor Chem imaging system (San Jose, CA) after immersing in enhanced chemiluminescence (ECL) solution (Solarbio, catalog: PE0010). The relative intensity of protein signals was normalized to the corresponding loading control intensity and quantified by Image J software.

### Gelatin Gel Zymography

Gelatin gel zymography was utilized on 6 h, 12 h, days 1, 3, and 5 post-ICH to measure the MMP-9 (Gelatinase B, 98 kD) gelatinolytic activity in the hemorrhagic brain as previously described (Li et al., 2017b). Protein samples were extracted from the ipsilateral brain area containing the striatum after the c-ICH and b-ICH models. Samples were loaded onto 10% Tris-tricine gels with 0.1% gelatin as a substrate and separated by electrophoresis. Following electrophoresis, gels were washed twice with 2.5% Triton X-100 for 1 h to remove the SDS and incubated 40 h at 37°C in digestion buffer (50 mM Tris–HCl, 50 mM NaCl, 5 mM CaCl_2_, 2 μM ZnCl_2_, 0.02% Brij-35, pH = 7.6). Then, the gel was stained with 0.5% wt/vol Coomassie blue R-250 for 2 h and then destained appropriately to be photographed. Bands were visualized with a gel-imaging system (BIO-RAD, United States), and band intensity was quantified with Image J analysis software.

### Quantification of Evans Blue Leakage

A solution of 2% Evans blue (EB) dye (4 mL/kg, Sigma Aldrich, St. Louis, United States) was slowly administered through the tail vein on 6 h, 12 h, days 1, 3, and 5 after ICH as previous describe (Wang et al., 2017; Yang et al., 2017). The mice were euthanized after a 3 h infusion and were then perfused transcardially with PBS. The brain tissue was removed, divided into right and left hemispheres, and weighed. Each part of the brain was homogenized in 1 mL of PBS and then sonicated and centrifuged (30 min, 9,424 g/min, 4°C) and the supernatant was collected. EB stain was measured by spectrophotometry at 620 nm and quantified according to a standard curve (Diluted with different concentrations of EB dye). The results are presented as the ratio of the left to right hemisphere.

### Measurement of Endogenous Immunoglobulin G (IgG) Extravasation

Immunohistochemical staining was performed according to previously reported methods (Mao et al., 2017). It was used to determine the area of extravasation of endogenous IgG molecules on days 3 and 5 in both ICH models. The mice were anesthetized and intracardially perfused with phosphate-buffered saline, followed by 4% paraformaldehyde. After dehydration, the brain was sliced into 20-mm-thick sections. Sections were blocked with 5% goat serum in phosphate-buffered saline with 0.1% Triton-X 100 for 1 h and were incubated in DyLight^TM^ 488-conjugated goat anti-rat IgG antibody (1:1,000; Jackson Immuno-Research Laboratories, West Grove, United States) at room temperature. Finally, stained sections were examined with a fluorescence microscope (Eclipse TE2000-E; Nikon, Japan).

### Transmission Electron Microscope

The BBB ultrastructure was scanned by transmission electron microscopy (TEM) on 6 h, 12 h, days 1, 3, and 5 after ICH (Li et al., 2017a, 2018). The mice were anesthetized and transcardially perfused with 0.9% saline, followed by a 0.1 M phosphate buffer (PB) containing 2.5% paraformaldehyde and 2% glutaraldehyde. The brain was extracted and divided into 1 mm three pieces and immersed in the same fixative at 4°C overnight. After being washed thoroughly with 7.5% sucrose buffer, samples were post-fixed with 1% osmium tetroxide, at 4°C for 2 h. The tissue proceeded to be block-stained with a 2% aqueous solution of uranyl acetate for 1 h. The tissue was dehydrated with a graded series of ethanol and embedded in acrylic resin. Serial ultrathin sections were cut with an ultramicrotome, stained with lead citrate and uranyl acetate, and observed with an electron microscope (HITACHI, HT7700, Japan). The length and number of the TJ were analyzed by using Image J software.

### Real-Time Polymerase Chain Reaction Analysis

Total mRNA was extracted from the brain tissues around the hematoma by QIAzol Lysis Reagent (miRNeasy Mini Kit; QIAGEN, Gaithersburg, MD) (Li et al., 2017b; Su et al., 2017). One thousand nanograms of RNA from each sample was transcribed into cDNA with the SuperScript VILO cDNA Synthesis Kit (Invitrogen, Carlsbad, United States). Reverse-transcribed RNA was amplified by PCR under the following conditions: 95°C for 5 min, 40 cycles of the 30 s at 94°C, 30 s at 65°C, 20 s at 72°C, and a final extension step for 10 min at 72°C. The quantitative polymerase chain reaction was performed with the Opticon 2 Real-Time Polymerase Chain Reaction Detection System (Bio-Rad, Hercules, United States) using corresponding primers and the SYBR green Polymerase Chain Reaction Master Mix (Applied Biosystems, Waltham, United States). The primer sequences that were utilized are listed in following:

AQP4:
5’- CTTTCTGGAAGGCAGTCTCAG -3’ (forward),
5’- CCACACCGAGCAAAACAAAGAT -3’ (reverse).
GAPDH:
5’- CAGTGGCAAAGTGGAGATTGTTG -3’ (forward),
5’- TCGCTCCTGGAAGATGGTGAT -3’ (reverse).

### Statistical Analysis

All data are reported as mean ± SD, dot plots, or bar graphs. One-way or two-way ANOVA and the Bonferroni *post-hoc* test were used to compare differences among multiple groups. All analyses were carried out with GraphPad Prism 5.0 Software. The criterion for statistical significance was *P* < 0.05.

## Results

### The Comparison of Injury Volume Between the c-ICH and the b-ICH Groups

**FIGURE 1:**
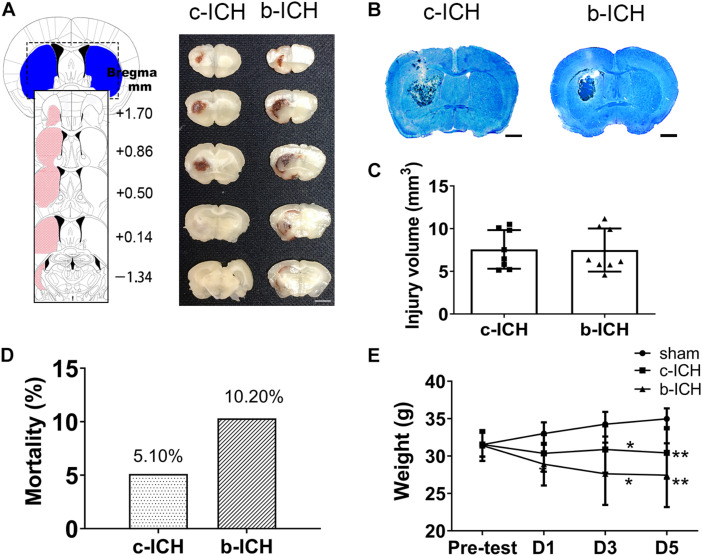
The comparison of injury volume between c-ICH and b-ICH groups. **(A)** Left: Schematic illustration of the spatial extent of the striatum. Right: Representative fresh brain sections show hematomas (red areas) at 24 h after intrastriatal injection of 0.050 U collagenase (“Collagenase”) or 20 μL autologous blood (“Blood”). **(B)** Representative Luxol fast blue/Cresyl violet-stained brain sections from c-ICH and b-ICH groups (scale bar = 1 mm). **(C)** Injury volume was detected at 24 h after ICH by integration of serial coronal sections stained with Luxol fast blue/Cresyl violet, no difference between the c-ICH and b-ICH model was detected. *n* = 8 mice/group (*t*-test). **(D)** Overall mortality was determined for all animals used in this study. **(E)** Bodyweight was measured in all groups on days 1, 3, and 5 respectively. *n* = 12 mice/group. *P < 0.05, **P < 0.01 vs. sham group (Repeated measures ANOVA followed by Bonferroni *post-hoc* test). Data are expressed as mean ± SD (the repeated measures ANOVA followed by the Bonferroni *post-hoc* test). Values are mean ± SD.

Injecting 20 μL autologous blood or 0.05 U collagenase VII into the left striatum resulted in a similar bleeding volume. Images of fresh brain slices illustrated that the cerebral hemorrhage was located in the striatum at 24 h (Figure 1A). LFB/CV staining slides portrayed the injury volume of the c-ICH and b-ICH group and was shown to be identical at 24 h after ICH (Figure 1B). The injury volume results further verified that hemorrhage volume had no statistical significance between the two groups (7.570 ± 2.264 mm^3^ in c-ICH groups vs. 7.493 ± 2.524 mm^3^ in b-ICH groups; *F* = 1.242, *n* = 8 mice/group, *P* > 0.05; Figure 1C). The total mortality of mice throughout the experiment differed between the experimental models in this study. No mice died in the sham group, but the mortality rate of the b-ICH group was shown to be higher than that of the c-ICH [10.20% (10 of 98) group vs. 5.10% (5 of 98) in the b-ICH group; Figure 1D]. Both ICH models were associated with a pronounced weight loss compared with the sham mice on days 3 and 5. However, there was no significant difference in the body weight between the c-ICH and b-ICH groups on days 1, 3, and 5 (*P* > 0.05; Figure 1E).

### The Comparison of Motor Function Between the c-ICH and the b-ICH Groups

**FIGURE 2:**
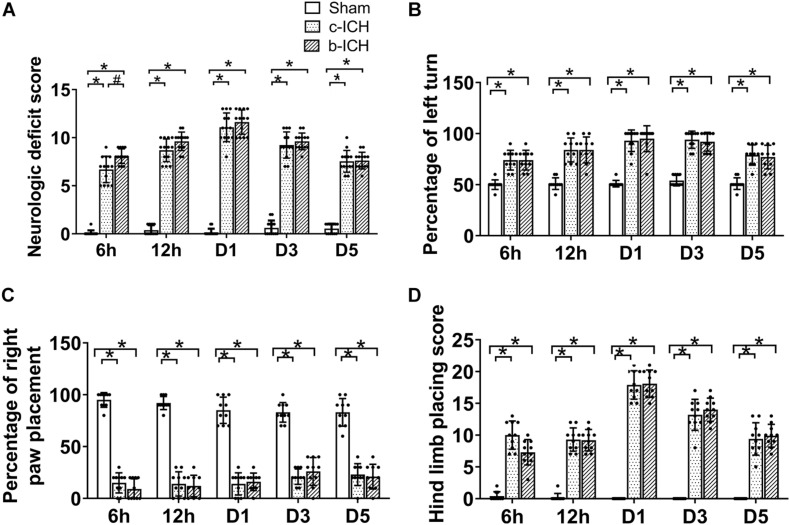
Motor function of the two ICH models. **(A)** Mice in the b-ICH group had a more severe neurologic deficit score (NDS) than the c-ICH group at 6 h after ICH. *n* = 10 mice/group. *P < 0.05 vs. sham group; ^#^*P* < 0.05 vs. ICH group (repeated measures ANOVA followed by Bonferroni *post-hoc* test). **(B–D)** The corner turn test and right front paw and hindlimb placement tests on 6 h, days 1, 3, and 5 after ICH. *n* = 10 mice/group. *P < 0.05 vs. sham group (the repeated measures ANOVA followed by the Bonferroni *post-hoc* test). Values are mean ± SD.

The NDS of the two model groups were significantly higher than those of the sham group which peaked on day 1 and proceeded to decrease gradually. The NDS of the b-ICH group was significantly higher than that of the c-ICH group only at 6 h after surgery (8.111 ± 0.261 in b-ICH groups vs. 6.833 ± 0.386 in c-ICH groups; *n* = 12 mice/group; *P* < 0.05). There was no significant difference at any other time points (*P* > 0.05, *n* = 12 mice/group; Figure 2A). Additionally, there were no significant differences in the results of the forelimb and hind limb placement and corner turn test between the two model groups at 6 h, 12 h, days 1, 3, and 5 (*n* = 10 mice/group; *P* > 0.05, Figures 2B–D).

### The Comparison of BBB Breakdown Between the c-ICH and the b-ICH Groups

**FIGURE 3:**
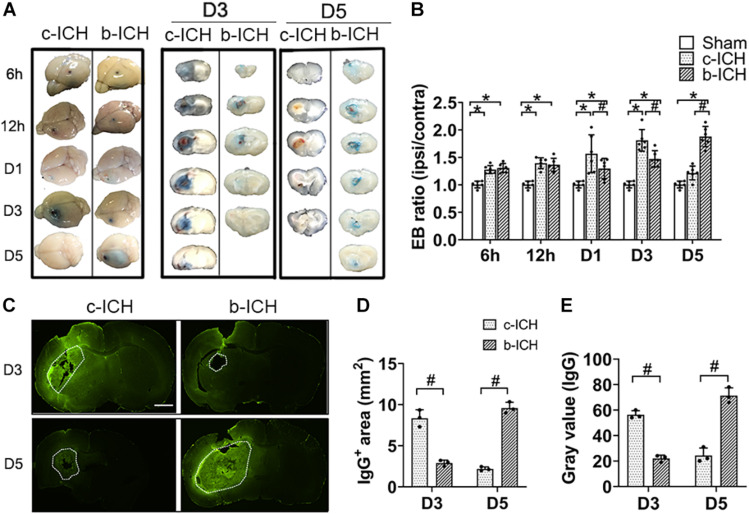
The comparison of BBB breakdown between the c-ICH and the b-ICH groups. **(A)** The Evans blue staining showed that BBB leakage was present from 6 h to day 5 after ICH in two groups. **(B)** Quantitative analysis showed that the Evans blue (EB) concentration peaked at day 3 in the c-ICH group but on day 5 in the b-ICH group (*n* = 6 mice/group for each time point; *P < 0.05 vs. sham group, ^#^*P* < 0.05 vs. the c-ICH group; repeated measures ANOVA followed by Bonferroni *post-hoc* test). **(C)** The IgG-positive area was determined by immunohistochemical staining on days 3 and 5, respectively (scale bar = 1 mm). **(D,E)** Quantification of endogenous IgG-positive area and gray values of IgG immunostaining; *n* = 3 mice/group; ^#^*P* < 0.05 vs. c-ICH group (*t*-test). Values are Mean ± SD.

The BBB breakdown was evaluated by EB staining and measurement of IgG extravasation. The EB dye can be combined with albumin (67 kD) in serum and therefore was utilized to measure the integrity of the BBB at 6 h, 12 h, days 1, 3, and 5 post-ICH. EB leakage was prominent on day 3 in the c-ICH group compared with that on day 5 in the b-ICH group (Figure 3A). The quantitative analysis further revealed that the ratio of EB concentration (ipsilateral/contralateral hemisphere) peaked on day 3 in the c-ICH group compared to that on day 5 in the b-ICH group (D3: 1.783 ± 0.392% in the c-ICH group vs. 1.472 ± 0.149 in the b-ICH group; D5: 1.216 ± 0.216% in the c-ICH group vs. 1.879 ± 0.815 in the b-ICH group; *n* = 6 mice/group; *P* < 0.05; Figure 3B). The EB staining data were further supported by the pronounced intrastriatal leakage of the plasma-derived IgG. The IgG leakage around the hematoma was more prominent in the c-ICH group than in the b-ICH group on day 3, whereas it was more prominent in the b-ICH group than in the c-ICH group on day 5 (*n* = 3 mice/group; *P* < 0.05; Figures 3C–E).

### The Comparison of the Changes of Tight Junction Expression Between the c-ICH and the b-ICH Groups

**FIGURE 4:**
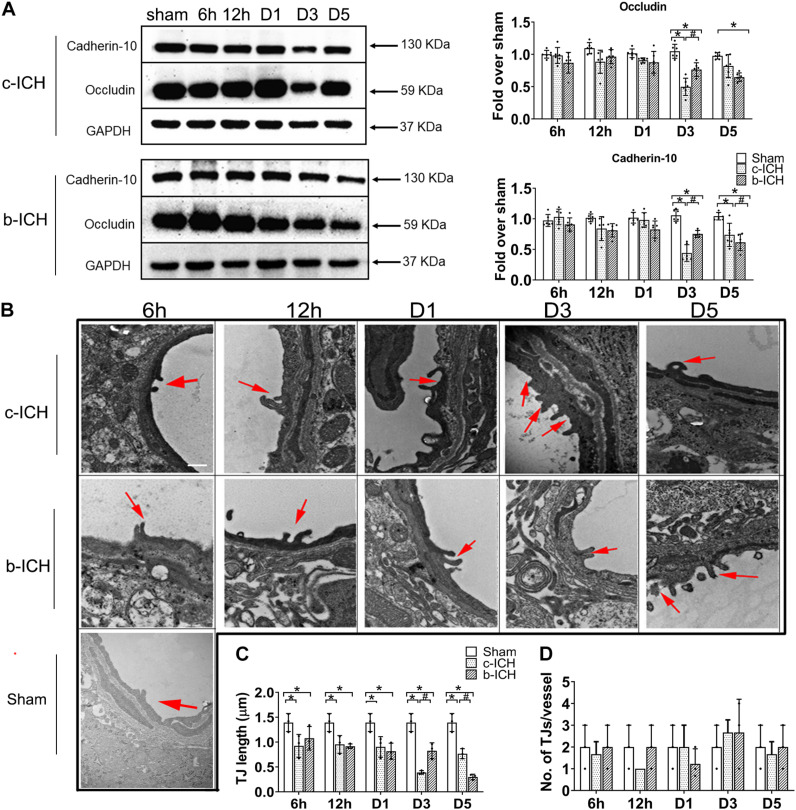
The comparison of the expressions of tight junction proteins between c-ICH and b-ICH groups. **(A)** Western blots showed occludin expression and cadherin-10 expression at 6 h, 12 h, days 1, 3, and 5 after ICH. Quantification of occluding and cadherin-10 levels at 6 h, 12 h, day 1, day 3, and day 5 after ICH in both models (*n* = 6 mice/group; *P < 0.05 vs. sham group, ^#^*P* < 0.05 vs. c-ICH group; repeated measures ANOVA followed by Bonferroni *post-hoc* test). Values are mean ± SD. **(B)** Representative electron microscopic images of TJ ultrastructure at 6 h, 12 h, day 1, day 3, and day 5 after ICH in both models and sham group. Arrowheads = tight junctions (TJ); Scale bar = 1 μM. **(C)** Quantification of tight junction length (*n* = 3/group). **(D)** Quantification of the number of tight junctions per vessel (*n* = 3 mice/group, four image fields quantified per mouse; *P < 0.05 vs. sham group, ^#^*P* < 0.05 vs. c-ICH group (the repeated measures ANOVA followed by the Bonferroni *post-hoc* test). Values are mean ± SD.

Endothelial cells (ECs) and their TJs are the primary components of the structure of the BBB. WB was used to detect the protein expression changes of TJs proteins including occludin and cadherin-10 in both ICH groups at 6 h, 12 h, days 1, 3, and 5 after ICH. The results revealed that the expression of occludin and cadherin-10 protein were significantly lower on day 3 in the c-ICH group (occludin: 0.498 ± 0.133% in the c-ICH group vs. 0.764 ± 0.106% in the b-ICH group; cadherin-10: 0.411 ± 0.136% in c-ICH group vs. 0.753 ± 0.058% in b-ICH group; *P* < 0.05; *n* = 6 mice/group) and on day 5 in the b-ICH group (occludin: 0.819 ± 0.178% in the c-ICH group vs. 0.647 ± 0.067% in the b-ICH group; cadherin-10: 0.738 ± 0.189% in the c-ICH group vs. 0.615 ± 0.133% in the b-ICH group; *P* < 0.05; *n* = 6 mice/group; Figure 4A). Reduction and the breaking of TJs in ECs after ICH was further confirmed by TEM. The ultrastructural changes of BBB were observed at 6 h, 12 h, days 1, 3, and 5 after ICH. Mice in the sham group showed intact and normal capillary endothelial cells as well as basal lamina. A high density of undamaged TJs was observed in the sham group (Figure 4B). However, BBB ultrastructural was dramatically damaged at the beginning of the 6th hour after ICH. The ECs were swollen, and TJs were shorter and blurred. Moreover, the basement membrane was thinner compared to the sham control (Figure 4B). Among them, the shortest width of TJs appeared on day 3 in the c-ICH group but on day 5 in the b-ICH group (day 3: 0.390 ± 0.036 in the c-ICH group vs. 0.827 ± 0.159 in the b-ICH group; day 5: 0.767 ± 0.10 in the c-ICH group vs. 0.298 ± 0.057 in the b-ICH group; *P* < 0.05; *n* = 3 mice/group; Figure 4C). There was no difference in the total numbers of TJs per vessel (*P* > 0.05; Figure 4D).

### The Comparison of the Expressions of Aquaporin 4 (AQP4) and MMP-9 Activity in Brain Tissue Between the c-ICH and the b-ICH Groups

**FIGURE 5:**
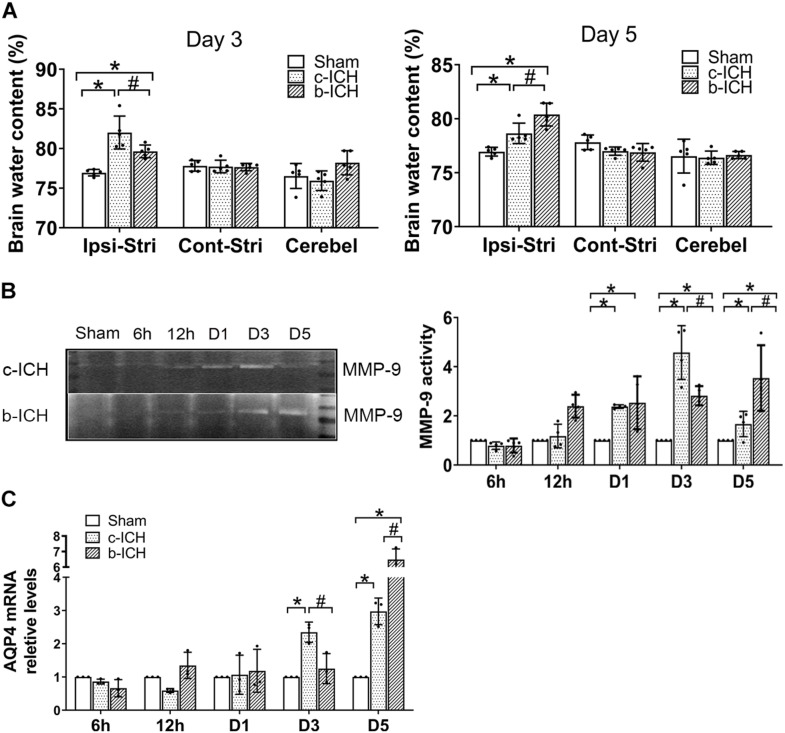
The comparison of the expressions of aquaporin 4 (AQP4) and MMP-9 activity in brain tissue between c-ICH and b-ICH groups. **(A)** Measurement of brain water content on days 3 and 5 (*n* = 6 mice/group; *P < 0.05 vs. sham group, ^#^*P* < 0.05 vs. the c-ICH group repeated measures ANOVA followed by Bonferroni *post-hoc* test). Cont-Stri, contralateral striatum; Ipsi-Stri, ipsilateral striatum. Values are mean ± SD. **(B)** Gelatin zymography shows MMP-9 activity in brain tissue at 6 h, 12 h, day 1, day 3, and day 5 after ICH in both models. Quantification of MMP-9 activity in the brain (*n* = 4 mice/group, *P < 0.05 vs. sham group, ^#^*P* < 0.05 vs. the c-ICH group; the repeated measures ANOVA followed by the Bonferroni *post-hoc* test). **(C)** Quantification of AQP4 mRNA levels in the brain at 6 h, 12 h, day 1, day 3, and day 5 after ICH in the c-ICH and the b-ICH groups (*n* = 3 mice/group, four fields quantified per mouse; *P < 0.05 vs. sham group, ^#^*P* < 0.05 vs. the c-ICH group; the repeated measures ANOVA followed by the Bonferroni *post-hoc* test). Values are mean ± SD.

Brain water content in the ipsilateral brain tissue in the c-ICH group was more severe than that in the b-ICH group on day 3 (82.012 ± 2.077% in the c-ICH group vs. 79.638 ± 0.803% in the b-ICH group; *P* < 0.05; *n* = 6 mice/group; Figure 5A) whereas it was more severe in the b-ICH group than that in the c-ICH group on day 5 (78.646 ± 0.944% in the c-ICH group vs. 80.392 ± 1.058% in the b-ICH group; *P* < 0.05; *n* = 6 mice/group; Figure 5B). The MMP-9 activity was detected by the gelatin zymography at 6 h, 12 h, days 1, 3, and 5 after ICH. The results showed that MMP-9 activity began to increase on day 1 after ICH in both models and it reached its peak on day 3 in the c-ICH group (4.575 ± 0.195% in the c-ICH group vs. 2.82 ± 0.39% in the b-ICH group; *P* < 0.05) and on day 5 in the b-ICH group (3.517 ± 0.337% vs. 1.668 ± 0.516%; *P* < 0.05; *n* = 6 mice/group; Figure 5B). At 6 h, 12 h, days 1, 3, and 5 after ICH, compared to the sham group, the relative expression of AQP4 mRNA began to increase on day 3 in the c-ICH group, and the expression was significantly higher than that in the b-ICH group (2.349 ± 0.305% vs. 1.251 ± 0.453%; *P* < 0.05). However, the relative expression of AQP4 mRNA was significantly higher in the b-ICH group than that in the c-ICH group on day 5 (6.491 ± 0.678% vs. 2.974 ± 0.399%; *P* < 0.05; *n* = 3 mice/group; Figure 5C).

## Discussion

In this study, we found that the BBB leakage was associated with a decrease in TJ protein expression and an increase in MMP-9 activity and AQP4 mRNA expression on day 3 in the c-ICH model but on day 5 in the b-ICH model. Additionally, utilizing TEM, we demonstrated that the ECs were swollen, and TJs were shorter and blurred after ICH compared to sham controls. Moreover, the basement membrane was thinner in the c-ICH model on day 3 compared to that in the b-ICH model on day 5. Therefore, combined with the characteristics of clinical ICH patients with locally elevated perihematomal permeability-surface area product (PS) derived from computed tomographic perfusion (CTP) imaging within 24 to 72 h after ICH (Xu et al., 2017), we conclude that the c-ICH model might be a more suitable model for studying early BBB damage and protection comparing to the b-ICH model.

The high morbidity and mortality of ICH promote the development of various ICH animal models in preclinical studies (Strbian et al., 2008; Li and Wang, 2017). It is speculated that a better model is the one that closely simulates the pathophysiology and functional consequences of ICH in humans. Infusions of autologous blood or collagenase are the most widely used rodent models to investigate pathophysiologic mechanisms and test experimental treatments after ICH (MacLellan et al., 2008). The b-ICH model that mimics a single intracerebral bleed can be applied to transgenic or knockout mice to study its specific signaling pathway or brain injury mechanism (Rynkowski et al., 2008). However, the model lacks underlying angiopathy and rupture (Wang et al., 2008). The c-ICH model utilizes enzymatic disruption of blood vessels to imitate spontaneous ICH. However, the induced bleeding is usually caused by the rupture of the large vessels, while the clinical ICH patients often have small deep penetrating artery rupture (Chu et al., 2004). During the operation of our experiment, we found that collecting enough blood quickly within 1 min is a key step to ensure the success of the b-ICH model.

In the early stage after ICH, the motor function of mice was slightly different between the c-ICH group and the b-ICH group. The mortality rate of b-ICH is higher than that of c-ICH (5.10 vs. 10.20%) in our study, which may be due to the direct space-occupying effect of brain tissue caused by the direct injection of hematoma pressure, increasing the death rate of animals. Based on our experience, the NSS is higher in the b-ICH group than that in the c-ICH group in the early time points (1–6 h) after ICH. A probable reason for this discrepancy may be that blood vessel damage by collagenase is a dynamic process, and consequently, blood accumulates and hematoma develops in the striatum gradually within 4–5 h (Liebner and Plate, 2010; Wang et al., 2015), which results in delayed neurologic deficits. The difference in early neurologic impairment cannot be detected by the other three behavioral tests, suggesting that the 24-point neurologic deficit scoring system is more sensitive for evaluating locomotor abnormalities, especially at early time points after ICH. The result is consistent with our previous research (Zhu et al., 2018). In the clinic, acutely increased intracranial pressure (ICP) after ICH is a life-threatening neurosurgical emergency (Leinonen et al., 2017). In addition, the dynamics of perihematomal edema (PHE) induced by BBB integrity occurs within hours after ICH, which has been implicated in secondary brain injury and could be a therapeutic target (Sprugel et al., 2019). In our research, we found that brain water content in the ipsilateral brain tissue in the c-ICH group was higher than that in the b-ICH group on day 3, whereas it was higher in the b-ICH group than that in the c-ICH group on day 5, indicating that both models can imitate the clinical PHE occurred after ICH. All inflammation, thrombin activation, and red blood cell lysis can lead to BBB destruction and edema formation, which has three stages: (1) clot retraction can force the serum into the surrounding space of hematoma and form angiogenic edema (1 h after ICH); (2) cytotoxic brain edema (peak D1-2) caused by inflammation and thrombin activation through coagulation cascade reaction; and (3) erythropoietin cytolysis and Hb toxicity related injury (delay) appeared edema on D3 (Zheng et al., 2016).

BBB, consisting of endothelial cells, pericytes, astrocytes, basement membrane, and extracellular matrix (Lv et al., 2018), is an important component of NVU that plays a crucial role in maintaining the homeostasis of the CNS but limits the number of potential therapeutic drugs capable of gaining access to the brain. Thus, it is important to study the characteristics of BBB injury for the treatment of nervous system diseases. We compared the BBB damage at 6 h, 12 h, 24 h, day 3, and day 5 after ICH between the two most common murine ICH models based on the similar injury volume. In our study, EB extravasation assay showed that an increase in leakage at 6 h in both models and the ratio of EB concentration (ipsilateral/contralateral hemisphere) peaked on day 3 in the c-ICH model whereas on day 5 in the b-ICH model, which was consistent with our previous study using the c-ICH model (Yang et al., 2017). In contrast to EB, the IgG (∼140 kD) extravasation was prominent around the hematoma on day 3 in the c-ICH group and day 5 in the b-ICH group after ICH. However, the exact mechanism of the IgG leakage after hemorrhage is still unclear. Transcellular mechanisms may contribute to IgG (∼140 kD) extravasation after ICH. FcRnh (The neonatal Fc receptor for IgG) and low-density lipoprotein receptor-related protein 1 (LRP1) that both expressed on BBB mediates the efflux of IgG across the BBB and facilitate IVIg internalization (Halliday et al., 2016).

MMPs are a group of important extracellular matrix-degrading enzymes, with various biologic functions. Thrombin, hemoglobin, cytokines, oxidative stress, hypoxia, and other factors can lead to increased expression/activity of MMPs (Wang, 2010). MMPs are a family of proteases that participate in physiologic and pathophysiologic processes, including at the BBB interface. It is shown that MMP-9 expression/activity is increased in the human ICH brain (Wu et al., 2010a). Once activated, MMPs disrupt the BBB by degrading tight junctions and basal lamina proteins, leading to BBB leakage and brain edema. We have reported in an early study that post-ICH application of GM6001 (a broad-spectrum MMP inhibitor) protected BBB and reduced brain edema, thereby mitigating neurologic deficits (Wang and Tsirka, 2005). Furthermore, knockout of the MMP-9 gene significantly decreases IgG accumulation in the parenchyma 24 h after neonatal H/I, suggesting that MMP-9 contributes to the early BBB opening. In addition, it is reported that Hb-induced oxidative stress after ICH may contribute to early BBB dysfunction and subsequent cell death through MMP activation (Katsu et al., 2010). Aquaporin-4 (AQP4) is expressed in pericapillary astrocyte foot processes and contributes to edema formation after ICH (Wu et al., 2010b). Prior research suggests that AQP4 deletion increases ICH damage, including edema formation, BBB damage, and neuronal death (Chu et al., 2014). There also is a significant increase in AQP4 expression in ICH patients (Dardiotis et al., 2019). In our research, by comparing MMP-9 enzymatic activity and APQ4 mRNA levels between the c-ICH model and the b-ICH model after ICH, we found that the MMP9 activity reached the peak on day 3 in the c-ICH group and on day 5 in the b-ICH group. Furthermore, the relative expression of AQP4 mRNA began to increase on day 3 in the c-ICH group while on day 5 in the b-ICH group. In conclusion, we are the first study to report that the severe BBB injury occurred on day 5 in the b-ICH model which has guiding value for future b-ICH research.

In this study, we examined the ultrastructural features of the mouse brain after collagenase- and whole blood-induced ICH. Both models share pathologic similarities in terms of basement membrane damage and TJ fracture at 6 h after ICH. However, the collagenase injection model does have the most serious TJ damage on day 3 and the blood injection model does have on day 5. Using TEM, we observed that the ECs were swollen, and TJs were shorter and blurred; furthermore, the basement membrane was thinner on day 3 in the c-ICH group and on day 5 in the b-ICH group, respectively. We also observed axonal demyelination and degeneration after ICH in both models.

## Conclusion

In conclusion, the progression of the BBB damage differs in the c-ICH and the b-ICH model; the BBB damage occurs earlier in the c-ICH model than that in the b-ICH model. The underlying mechanism of BBB breakdown is similar between these two ICH models. Combined with the characteristics of clinical ICH with a prominent increase in BBB leakage in the perihematomal regions of the patients with spontaneous basal ganglia ICH within 24–72 h after symptom onset (Li et al., 2018), we can conclude that the c-ICH model might be a more suitable model for studying early BBB damage and protection than the b-ICH model.

## Data Availability Statement

The original contributions presented in the study are included in the article/Supplementary Material, further inquiries can be directed to the corresponding author/s.

## Ethics Statement

The animal study was reviewed and approved by the Animal Ethics Committee of Zhengzhou University and followed the ARRIVE guidelines.

## Author Contributions

PJ performed the experiments and wrote and revised the manuscript. XC and JiW supervised the research and wrote and revised the manuscript. All the authors participated in commenting on and approving the final manuscript.

## Conflict of Interest

The authors declare that the research was conducted in the absence of any commercial or financial relationships that could be construed as a potential conflict of interest.

## Publisher’s Note

All claims expressed in this article are solely those of the authors and do not necessarily represent those of their affiliated organizations, or those of the publisher, the editors and the reviewers. Any product that may be evaluated in this article, or claim that may be made by its manufacturer, is not guaranteed or endorsed by the publisher.

## Footnotes

1
http://www.nc3rs.org.uk/arrive-guidelines

2
www.randomization.com

## Supplementary Material

Supplementary Figure 1 Experimental design route. NFE, Neurologic function evaluations; EB, Evans’s blue; WB, Western blot; TEM, Transmission electron microscope; GEZ, Gelatin gel zymography; CV-LFB, cresyl violet and Luxol fast blue; HIA, Hemorrhagic injury analysis; IgG ET, IgG extravasation test.

## Abbreviations

AQP4: aquaporin 4
BBB: blood brain barrier
BCA: bicinchoninic acid
BMECs: micro-vascular endothelial cells
BWC: brain water content
CV-LFB: cresyl violet and Luxol fast blue
c-ICH: collagenase intracerebral hemorrhage model
b-ICH: autologous blood intracerebral hemorrhage model
EB: Evans blue
ECL: enhanced chemiluminescence
ECs: endothelial cells
ICH: intracerebral hemorrhage
MMP9: matrix metalloproteinases 9
NDS: neurological deficit scores
PB: phosphate buffer
PVDF: polyvinylidene fluoride
SDS-PAGE: sodium dodecyl sulfate polyacrylamide gel electrophoresis
SPF: specific pathogen-free
TEM: Transmission electron microscope
TJ: tight junction
WB: western blotting.
