# Supplementary figures and images for: Profiling of Blood-Brain Barrier Disruption in Mouse Intracerebral Hemorrhage Models: Collagenase Injection vs. Autologous Arterial Whole Blood Infusion
Source: Front Cell Neurosci. 2021 Aug 26;15:699736. doi: 10.3389/fncel.2021.699736 (PMC8427528; doi:10.3389/fncel.2021.699736)

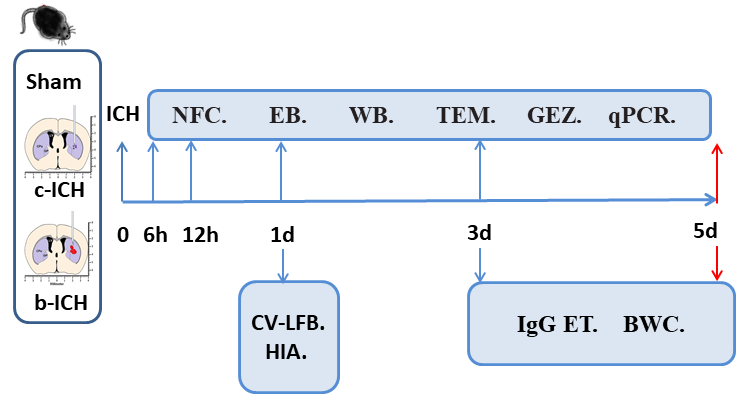

Supplement: Supplementary Figure 1 — Experimental design route. NFE, Neurologic function evaluations; EB, Evans’s blue; WB, Western blot; TEM, Transmission electron microscope; GEZ, Gelatin gel zymography; CV-LFB, cresyl violet and Luxol fast blue; HIA, Hemorrhagic injury analysis; IgG ET, IgG extravasation test. [file Image_1.jpeg]
